# Do weight perception and bullying victimization account for links between weight status and mental health among adolescents?

**DOI:** 10.1186/s12889-021-11037-8

**Published:** 2021-06-04

**Authors:** Karen A. Patte, Maram Livermore, Wei Qian, Scott T. Leatherdale

**Affiliations:** 1 Department of Health Sciences, Brock University, Niagara Region, 1812 Sir Isaac Brock Way, St. Catharines, Ontario L2S 3A1 Canada; 2 School of Public Health and Health Systems, University of Waterloo, 200 University Ave, Waterloo, Ontario N2L 3G1 Canada

**Keywords:** Youth, Depression, Anxiety, Obesity, Internalizing symptoms, Overweight, Adolescents, Weight perception

## Abstract

**Background:**

The purpose of this study was to explore whether the way youth perceive their weight and their experiences of bullying victimization account for the increased risk of depression and anxiety symptoms, and poor psychosocial well-being, associated with overweight/obesity in a large sample of Canadian secondary school students. We also explored if associations differed by gender.

**Methods:**

We used cross-sectional survey data from year 7 (2018–19) of the COMPASS study. The sample included 57,059 students in grades 9–12 (Secondary III-V in Quebec) at 134 Canadian secondary schools (Alberta, British Columbia, Ontario, Quebec). First, multiple regression models tested associations between body mass index (BMI) classification and mental health outcomes (anxiety [GAD-7] and depression [CESD-10] symptoms, and psychosocial well-being [Diener’s Flourishing Scale]). Second, weight perception and bullying victimization were added to the models. Models were stratified by gender and controlled for sociodemographic covariates and school clustering.

**Results:**

When weight perception and bullying victimization were added to the models, obesity BMI status no longer predicted internalizing symptoms and flourishing scores relative to normal-weight BMIs. Students with ‘overweight’ or ‘underweight’ perceptions, and experiences of bullying victimization in the past month, reported higher anxiety and depressive symptomatology, and lower flourishing levels, in comparison to students with ‘about right’ weight perceptions and without experiences of bullying victimization, respectively, controlling for BMI status. Results were largely consistent across boys and girls.

**Conclusions:**

Results suggest perceptions of weight and experiences of bullying independently contribute to differences in mental health outcomes by weight status among youth. Continued efforts targeting weight-based bullying and weight bias, and the promotion of body size acceptance and positive body image, may help reduce the risk of mental illness and poor mental health among adolescents.

## Introduction

About one in seven adolescents are considered at-risk of overweight and obesity [1]. Obesity is associated with elevated rates of depression and anxiety [2, 3], two of the most common forms of mental illness in children and adolescents [4]. Nearly one-third of adolescents will experience an anxiety disorder and about 12% will have major depressive disorder [4]. Given their prevalence, chronicity, and adverse impacts, obesity and mental disorders have been identified as global public health priorities. More recently, the importance of promoting positive mental health, in addition to preventing mental illness, has been acknowledged [4]. Further research on the complex relationship between weight status and mental health among adolescents is needed.

The adverse consequences commonly associated with overweight and obesity in children and adolescents are primarily psychosocial [5–8], and include low self-esteem, poorer social functioning, and greater risk of depression and anxiety [2, 5, 9–11]. Psychological consequences of obesity may be attributable to weight stigmatization and discrimination [8, 11–13]. Weight stigma refers to the social de-evaluation/degradation of a person because of their weight through negative attitudes or beliefs and often manifests in ways that lead to social rejection and overt discrimination [7]. In children and adolescents, weight stigma is primarily expressed through teasing and bullying [8]; adolescents identify weight as the primary reason for harassment among their peers [14, 15]. Youth with overweight and obesity are more likely to experience bullying victimization than their average-weight counterparts, including relational, verbal, and physical victimization [16–18]. Associations between weight-based teasing or bullying victimization and increased vulnerability to depression, anxiety, substance use, psychosomatic symptoms, and low self-esteem, persist after controlling for body mass index (BMI) [19–21], leading to suggestions that experiences of stigma rather than weight itself contribute to negative outcomes [8, 19].

In addition to public stigma (i.e. perceived stigma and experiences of weight discrimination, including negative attitudes from peers and inappropriate person-to-person behaviours), individuals identifying with stigmatized groups can apply negative stereotypes to themselves [8, 22]. Adolescents who perceive themselves as overweight may be at risk of internalizing weight stigma, and in turn, experience low self-esteem and psychological distress [22]. Growing evidence suggests that weight perception (WP), rather than weight itself, largely accounts for many adverse psychosocial outcomes commonly associated with obesity [10, 23–27]. WP refers to how an individual evaluates or ‘sees’ their weight status and can be influenced by sociocultural weight norms and body ideals [28]. Studies suggest that psychological distress, lower psychological or social functioning, and depression and anxiety symptoms are better predicted by perceptions of being overweight than actual weight status [10, 23, 26, 29, 30]. In some research, the psychosocial risks associated with overweight/obesity by BMI status were no longer significant after controlling for WP [10, 23, 26, 29, 30].

The psychological connotations of weight status and WP may shift with changes in sociocultural norms. In UK adolescents, comparing 2015 to 1986 and 2005 estimates, behaviours aimed at weight loss and overestimation of weight status increased over time, adjusting for BMI, and in girls, weight loss behaviors were associated with greater depressive symptoms in 2015 compared to previous decades [31]. Similarly, in a nationally representative study of US adolescents, from 1999 to 2001 to 2015–2017, the risk of suicidality associated with overweight perceptions increased in recent years [32]. The authors attribute the difference to increased obesity stigma. Previous US studies have found increases in perceived weight discrimination in adults from 1995 to 1996 through 2004–2006 [33] and declines in social acceptance of people with obesity from 2013 to 2014 [34]. Against this backdrop, we have seen movements towards preventing weight bias and bullying, and promoting size acceptance and positive body image [35–37]. Some evidence also points to variations by geographic location, with stronger adverse effects of overweight perceptions on mental health in North American than Asian samples [29]. Existing peer-reviewed literature on WP and mental health that controlled for weight status, is predominantly from the US [27, 30, 32, 38], UK [39], Australia [25], China [40, 41], or Netherlands [10, 26], while research among Canadian adolescents remains limited.

Further work is also needed to clarify gender differences in the relationships among mental health, weight status, WP, and bullying. Existing evidence is mixed, with many studies not reporting on sex or gender [9]. In general, females and girls/women are at greater risk of psychological distress [42] and internalizing disorders, including generalized anxiety and major depression [40, 41]. Some literature indicates the social and emotional consequences of overweight/obesity are worse and occur at lower BMIs for girls/women than boys/men [9, 20]. In a systematic review including 19 studies, weight-based teasing was more common and had stronger associations with depressive symptoms among girls than boys [20]. Other studies have found the reverse; in US youth, the effect of perceived overweight on depression risk was stronger in males than females [30]. Gender differences may emerge in adolescence, with increased importance attributed to appearance, peer acceptance, and attainment of various sociocultural body ideals of thinness versus muscularity and leanness, and sex differences in pubertal development [28]. A recent UK longitudinal study found overweight perceptions in females only, and underweight perceptions in both males and females, predicted clinically relevant internalizing symptoms among adolescents with normal-weight BMIs [39]. In Canadian youth, in unadjusted descriptive findings, a smaller proportion of boys who thought their body was “too fat” or “too thin” had high emotional well-being scores relative to those who reported their weight was “normal” [43]. In girls, a smaller proportion with “too fat” perceptions had high emotional well-being, while those with “too thin” perceptions resembled those with “normal” perceptions [43].

Research on underweight perceptions or BMI-derived underweight status is more limited and mixed than literature on overweight and obesity. Perceptions of deviations from the social norm or body ideals in either direction present risk [23, 26, 39]. Underweight perceptions have been associated with depression and anxiety in males [24, 44, 45], and increased odds of psychological distress, internalizing symptoms, and suicidality in all youth [23, 25, 26, 39]. Some studies indicate underweight youth are more likely to be victims of bullying [18, 46], while others found no difference [46, 47] relative to their normal-weight peers. In a cross-national study including 39 countries, both underweight status and perceptions of being “too thin” were associated with greater risk of bullying victimization than normal weight status and perceptions, albeit to a lesser degree relative to obesity and “too fat” perceptions [48].

A clearer understanding of the roles of weight perception and bullying victimization in mental health and ill-health outcomes among adolescents across body weights will help inform intervention and primary prevention programs. In a large Canadian adolescent sample, the current paper aimed to explore whether associations between weight status and psychosocial well-being and internalizing symptoms (depression and anxiety) persist when controlling for weight perception and bullying victimization. It was hypothesized that perceptions of overweight or underweight, and experiences of bullying victimization, would independently predict higher depressive and anxiety symptomatology and poorer psychosocial well-being, regardless of weight status. Associations between weight status and mental health outcomes were expected to be reduced when controlling for weight perceptions and bullying victimization. Gender differences were also explored, with the hypothesis that overweight perceptions would present greater risk in girls, while underweight perceptions would be more common and detrimental in boys, given societal body ideals of masculinity and muscularity, and femininity and thinness.

## Methods

### Design

The Cannabis use, Obesity, Mental health, Physical activity, Alcohol use, Smoking, and Sedentary behaviour (COMPASS) Study is an ongoing prospective study designed to collect hierarchical health data once annually from students in grades 9 through 12 (Secondary I-V in Quebec) and the secondary schools they attend [49]. School boards and schools were purposefully selected based on whether they permitted active-information passive-consent parental permission protocols [36], which are critical for collecting robust youth data [50, 51]. Using these procedures, parents were informed of the study and were told to contact the COMPASS research coordinator if they did not want their child to participate. All students attending participating schools were eligible to participate, providing COMPASS staff did not hear from their parent/guardian to withdraw them. Eligible students willing to participate provided their informed assent and completed surveys during class time. Students could withdraw participation at any time. Further details of COMPASS methods are available online (www.compass.uwaterloo.ca) or in print [49]. All procedures were approved by the University of Waterloo (ORE#17264, #30118) Office of Research Ethics, Brock University Office of Research Ethics Board (REB#18–099), and appropriate school board committees, and were conducted in accordance with the Tri-Council Policy Statement: Ethical Conduct for Research Involving Humans – TCPS 2 (2018).

### Measures

#### Depression symptoms

Depressive symptoms were measured using the 10-item *Center for Epidemiologic Studies Depression scale Revised (CESD-10)* [52–54]. Items assess characteristics of clinical depression, including negative affect, anhedonia, and somatic symptoms, such as “I felt everything I did was an effort”, “I could not get ‘going’”, difficulty concentrating, restless sleep, and feelings of loneliness and hopelessness. Students were asked how often they experienced each symptom within the last 7 days, with the response options: “None or less than 1 day”, “1–2 days”, “3–4 days”, or “5–7 days”. Responses were scored from 0 to 3, respectively, and summed (plausible range 0–30). Two items assessing positive affect were reverse scored. Higher total scores indicated greater depressive symptoms. The scale has demonstrated validity in adolescents [55–58]. The internal consistency was good (α = 0.76).

#### Anxiety symptoms

Anxiety symptoms were measured using the 7-item Generalized Anxiety Disorder scale (GAD-7) [59]. The seven items consist of symptoms such as “feeling nervous, anxious, or on edge”, restlessness, uncontrollable worry, “becoming easily annoyed or irritable”, and “trouble relaxing”. Students were asked how often they experienced each symptom in the last 2 weeks, with response options: “Not at all”, “several days”, “over half the days”, or “nearly every day”. Responses were scored from 0 to 3 respectively and summed, for possible score range of 0–21. The GAD-7 has demonstrated validity in clinical and general population samples of adolescents [60–62]. Internal consistency was high in this sample (α = 0.91).

#### Flourishing

Diener’s 8-item Flourishing Scale (FS) [63] was used to measure self-perceived psychosocial functioning and well-being. Items assessed how youth perceived their relationships, life purpose and satisfaction, engagement and interest with daily activities, optimism, self-esteem and competence, to provide a total score that reflects overall psychosocial well-being. The original 7-point Likert scale was simplified to a 5-point Likert scale (1 = strongly disagree to 5 = strongly agree), by omitting the “slightly agree” and “slightly disagree” options, to be suitable for large school-based surveys among youth. Possible sum scores range from 8 to 40, on a languishing-flourishing continuum, with all item statements positively framed. The FS has demonstrated validity in youth populations [64, 65], and showed measurement invariance for gender, grade, and ethnicity in the current study [66]. The FS had a high internal consistency (α = 0.90).

#### Weight status

Body mass index (BMI; kg/m^2^) classifications (recoded as underweight, normal-weight, overweight, obesity, missing) were based on previously validated student-reported height and weight measures [67], and the World Health Organization age- and sex-adjusted cut-points. Missing BMI was included as category, due to the high amount of missing data (primarily due to missing weight, followed by height) that cannot be excluded as missing completely at random [68].

#### Weight perception (WP)

Consistent with previous studies [25, 38, 68], subjective perceptions of weight were assessed by asking “how do you describe your weight?” Response options included: “very underweight,” “slightly underweight,” “about the right weight,” “slightly overweight,” and “very overweight”. Responses were collapsed into 3 categories: very/slightly underweight, about right, and very/slightly overweight. As with weight status, missing WP was included as a fourth category.

#### Bullying victimization

Students were asked whether they were victims of bullying. Students who indicated: “I have not been bullied in the last 30 days,” were considered “not bullied” and all other students were considered to have experienced bullying victimization.

#### Covariates

Student-level covariates included grade (9, 10, 11, 12) and student-identified race-ethnicity (White, Black, Asian, Latinx, other/mixed). School postal codes were cross-referenced with Statistics Canada data to determine school-area median average income (≤50,000; 50,001-75,000, 75,001-100,000, > 100,000) and urbanicity (Rural, and Small, Medium, and Large Urban) of the area.

### Sample

Cross-sectional student data were used from the most recent complete wave of the COMPASS Study where data were collected prior to the COVID-19 pandemic (Year 7 [2018–2019]). In Year 7, data were collected from 74,501 students (84.2% participation rate) attending 134 secondary schools (8 Alberta, 15 British Columbia [BC], 61 Ontario, 52 Quebec). Missing students primarily resulted from student absenteeism and spare study periods during data collection. For consistency in participant ages across province, only students who would fall into the typical North American secondary school grades of 9–12 (International Standard Classification of Education level 3; corresponding to 13–18 years of age) were included in analyses, excluding 4406 girls and 4407 boys in secondary I or II in Quebec (equivalent to grades 7 and 8) and students in a class with no official grade equivalent (such as “new immigrant” classes offered in Quebec). Quebec students in secondary III through V were classified as grade 9 through 11 respectively, where secondary education ends in the province. Students missing responses on the outcome scales were removed from the models, resulting in the removal of 3868, 1306, and 1995 girls, and 4494, 1794, and 2353 boys, for missing data on the CESD-10, FS, and GAD-7 scales, respectively. The final analytic sample included 57,059 students. Students missing WP and data to determine BMI were retained in the sample.

### Statistical analysis

All analyses were implemented in SAS 9.4. Descriptive statistics were calculated for male and female students using the procedures PROC FREQ and PROC TTEST. Multiple regression models were tested using, with school clustering specified to account for within-school correlations. The intra-class correlation coefficient (ICC) was calculated for each outcome, to determine level of clustering present. The ICCs were 0.026, 0.016, and 0.019 for the FS, GAD-7, and CESD-10 scales, respectively, suggesting that between 1.6 and 2.6% of the variance was explained at the school-level. First, regression models tested associations between student BMI classification and the three mental health outcomes (anxiety [GAD-7] and depression [CESD-10] symptoms, and psychosocial well-being [FS]). Second, weight perception and bullying victimization were added as independent variables to the models. All models controlled for sociodemographic covariates (student grade and race-ethnicity, and school median household income and urbanicity), and were stratified by gender Significance was considered *p* < 0.001 given the large sample size and highly powered analysis.

## Results

### Descriptive statistics

See Table 1 for sample descriptives by gender. Students were about equally distributed across grades 9–11, with fewer in grade 12, as Quebec grades of Secondary I-V correspond to grades 7–11. Over 60% of the sample reported white race/ethnicity. More girls reported overweight perceptions (27.5%) than boys (21.1%) (*p* < 0.0001), yet more boys had BMIs considered overweight (13.8%) or obese (7.6%) than girls (10.6%; 4.2%) (p < 0.0001). Approximately twice the proportion of boys reported underweight perceptions (21.6%) than did girls (10.7%) (*p* < 0.0001), while only 1.3 and 2.1% of girls and boys respectively had underweight BMIs. About one-quarter of students were missing BMI data due to nonresponse to height, weight, and/or age. Girls had higher depressive and generalized anxiety symptoms relative to boys, yet lower flourishing scores (all p < 0.0001). One-fifth of boys and girls reported having experienced bullying victimization in the last 30 days.

**Table 1:** Descriptive statistics among Canadian secondary school students participating in Year 7 (2018/19) of the COMPASS Study

	Girls	Boys
	N = 28,580	***N*** = 28,479
	% (N)	% (N)
Grade		
9	27.9 (7984)	27.6 (7849)
10	28.2 (8047)	27.9 (7950)
11	26.7 (7641)	26.5 (7558)
12	17.2 (4908)	18.0 (5122)
Race/Ethnicity		
White	64.1 (18314)	62.6 (17814)
Black	3.2 (924)	4.5 (1294)
Asian	13.9 (3959)	13.9 (3954)
Latinx	2.6 (746)	2.8 (793)
Other/Mixed	16.3 (1637)	16.2 (4624)
BMI Classification		
Underweight	1.3 (384)	2.1 (588)
“Normal-weight”	58.5 (16722)	52.2 (14856)
Overweight	10.6 (3037)	13.8 (3927)
Obesity	4.2 (1198)	7.6 (2155)
Missing	25.3 (7239)	24.4 (6953)
Weight Perception		
Underweight	10.7 (3070)	21.6 (6147)
“About the right weight”	60.3 (17228)	55.4 (15777)
Overweight	27.5 (7867)	21.1 (6021)
Missing	1.5 (415)	1.9 (534)
Bullying Victimization (in the last 30 days)		
Yes	20.3 (5814)	20.2 (5757)
No	79.7 (22766)	79.8 (22722)
School-area median household income		
$25,000–50,000	11.2 (3210)	10.5 (2991)
$50,001–75,000	55.7 (15908)	56.0 (15960)
$75,000–100,000	27.3 (7798)	27.6 (7872)
> $100,000	5.8 (1664)	5.8 (1656)
Urbanicity		
Large Urban	58.6 (16740)	59.0 (16789)
Medium Urban	13.9 (3986)	13.8 (3938)
Small Urban	27.2 (7777)	26.9 (7675)
Rural	0.3 (77)	0.3 (77))
	**Mean (SD)**	**Mean (SD)**
Depressive symptoms (CESD-10)	10.2 (6.4)	7.6 (5.4)
Anxiety symptoms (GAD-7)	8.1 (5.9)	4.8 (5.1)
Flourishing (Flourishing Scale [FS])	31.3 (5.7)	32.1 (5.7)

NOTES: Possible score ranges include 0–30 for the CESD-10, 0–21 for the GAD-7, and 8–40 for the FS. Scores of ≥10 for the CESD-10 and GAD-7 are established cut-offs indicating clinically relevant symptoms. Secondary III, IV, and V in Quebec were classified as grades 9, 10, and 11, respectively

**Fig. 1:**
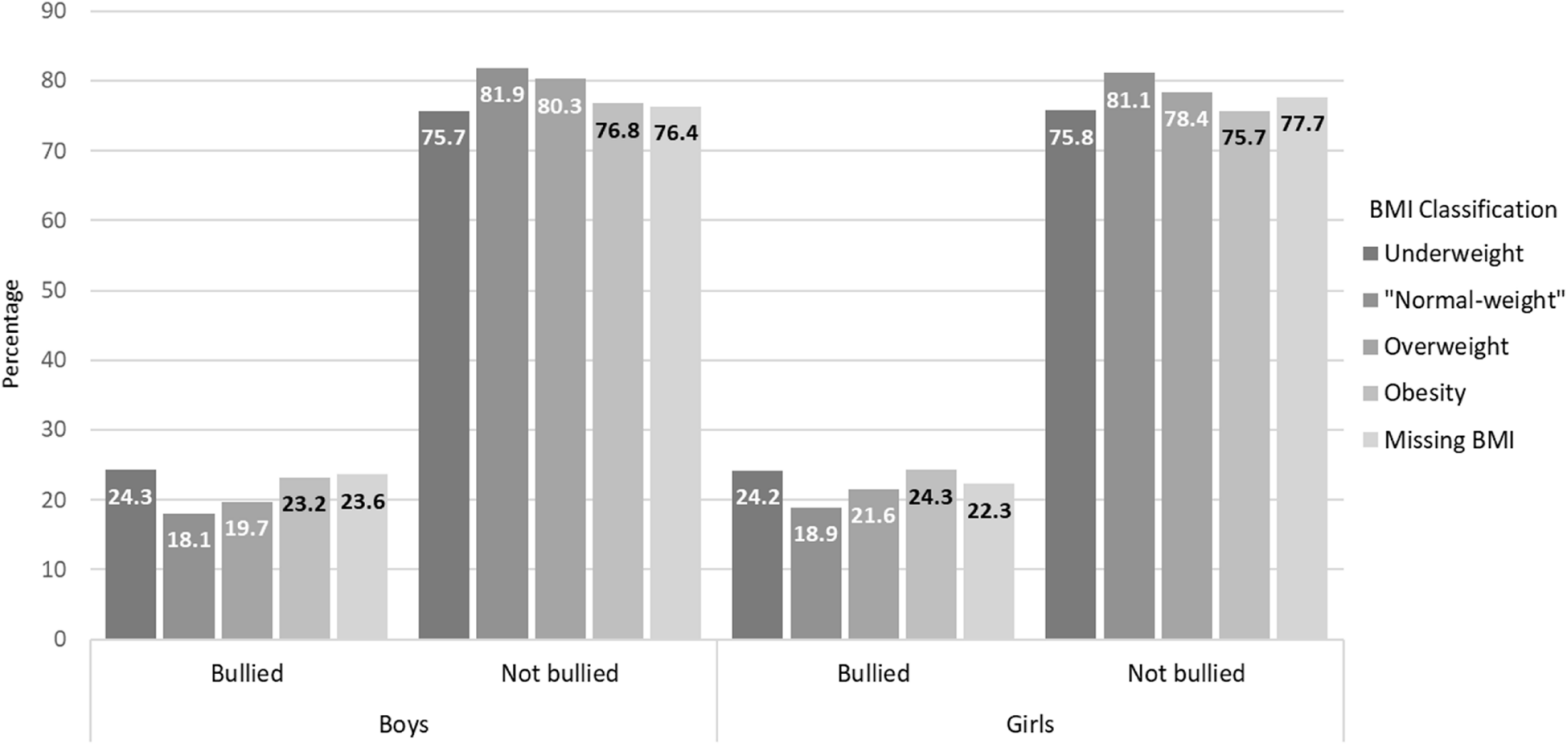
Self-reported bullying victimization within the last 30 days by BMI classification among boys and girls in Year 7 (2018/19) of the COMPASS Study

**Fig. 2:**
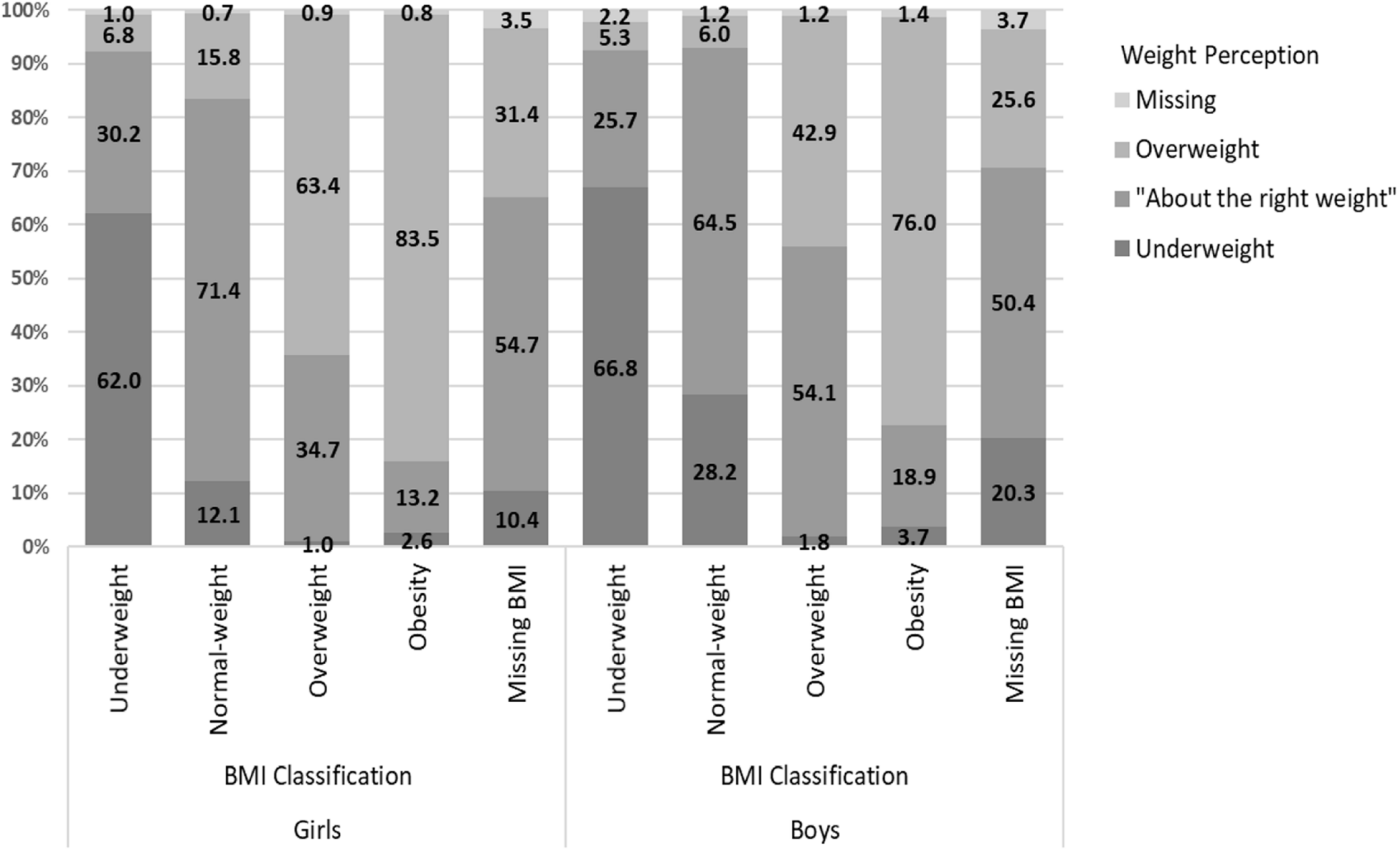
Weight perception by BMI classification among boys and girls in Year 7 (2018/19) of the COMPASS Study

**Fig. 3:**
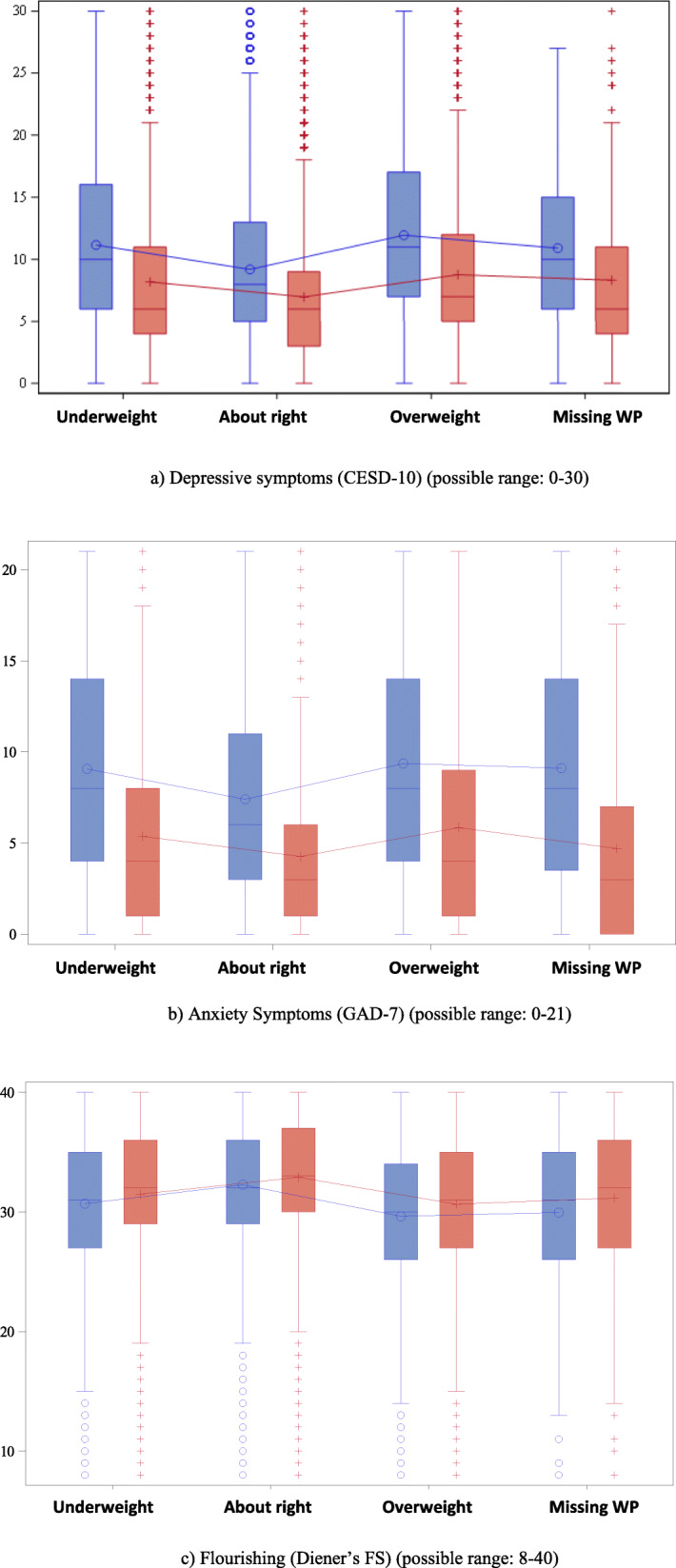
Depression, anxiety, and flourishing scores by weight perception among girls (blue) and boys (red) in Year 7 (2018/19) of the COMPASS Study. (**a**) Depressive symptoms (CESD-10) (possible range: 0–30). (**b**) Anxiety symptoms (GAD-7) (possible range: 0–21). (**c**) Flourishing (Diener’s FS) (possible range: 8–40). Notes: Higher scores indicate greater depressive and anxiety symptoms, and lower flourishing

**Fig. 4:**
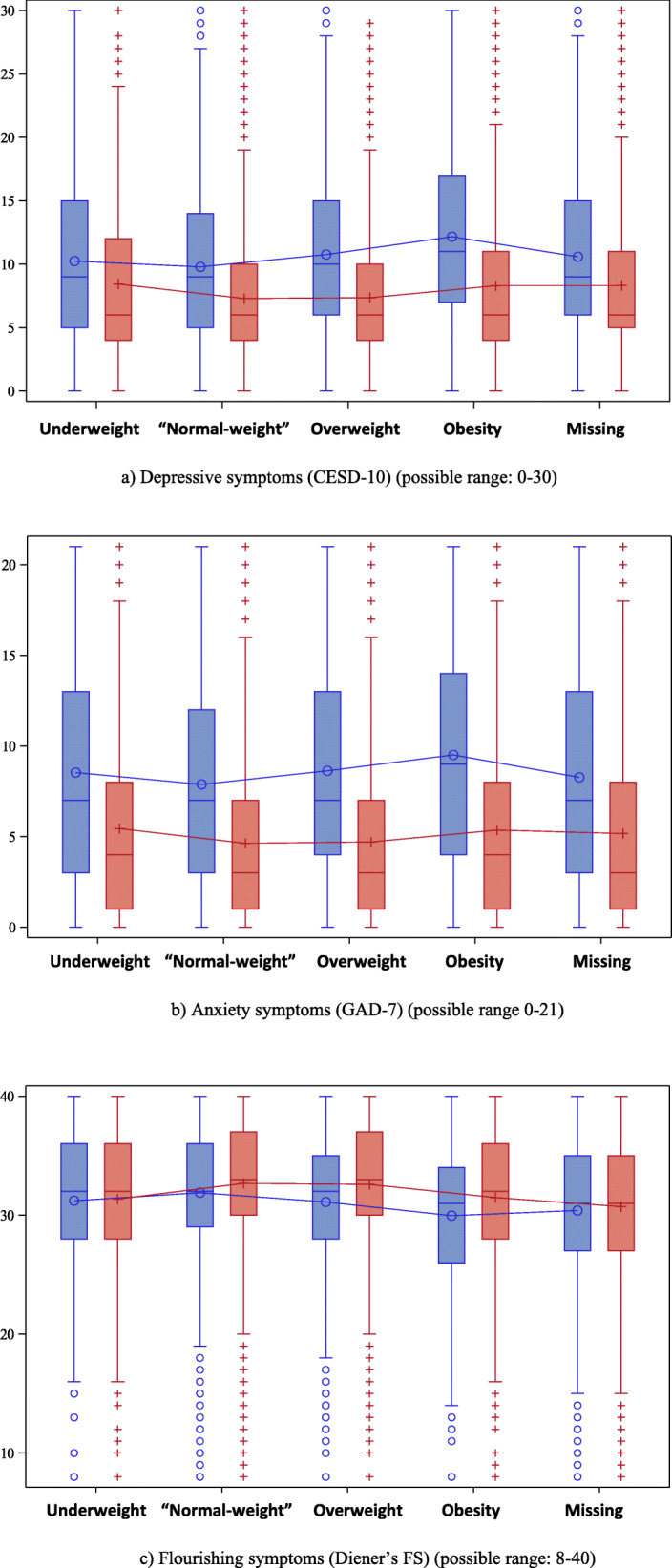
Depression, anxiety, and flourishing scores by BMI classification among girls (blue) and boys (red) in Year 7 (2018/19) of the COMPASS Study. (**a**) Depressive symptoms (CESD-10) (possible range: 0–30). (**b**) Anxiety symptoms (GAD-7) (possible range: 0–21). (**c**) Flourishing (Diener’s FS) (possible range: 8–40). Notes: Higher scores indicate greater depressive and anxiety symptoms, and lower flourishing

Figure 1 displays bullying victimization by BMI category in boys and girls. Bullying victimization was reported by the lowest proportion of girls with normal-weight BMIs (18.9%), and highest in those with BMIs in the underweight (24.2%) and obesity (24.3%) ranges. Among boys, bullying victimization was also reported by the lowest proportion with normal-weight BMIs (18.1%), but was highest in those with underweight BMIs (24.3%), followed closely by boys with BMIs in the obesity range (23.3%), while a relatively lower proportion of those with overweight BMIs (19.7%) reported bullying. Figure 2 displays weight perception by BMI classification in girls and boys. Across all BMI classifications, over half of students had concordant WPs, and more boys reported underweight WPs while girls more often reported overweight perceptions. Figure 3 displays the mental health measures by WP in girls and boys, and Fig. 4 displays the mental health measures by weight status.

### Regression models

#### Girls

The results of the regression models in girls are presented Table 2. The first three models tested associations between BMI classification and depression and anxiety symptoms, and flourishing scores, adjusting for covariates (student grade and race/ethnicity, and school area median household income and urbanicity) and school clustering. Girls with BMIs in the overweight or obesity range had a higher risk of depressive and generalized anxiety symptoms, and lower flourishing scores, than their peers with BMIs in the normal-weight range. Girls with missing BMI data were also at higher risk of depression symptoms and lower flourishing scores, but did not differ on anxiety symptoms from normal-weight girls. Girls with underweight BMIs did not differ from their peers with normal-weight BMIs. When WP and bullying were added to the models, BMIs classifications of overweight and obesity were no longer significantly associated with depression, anxiety, or flourishing; however, the associations with missing BMI remained significant.

**Table 2:** Weight status (BMI classification), weight perception (WP), and bullying victimization as predictors of depression, anxiety, and flourishing scores among **girls** in Year 7 (2018/19) of the COMPASS Study

	BMI			BMI + WP			BMI + WP + Bullying		
	Est.	95% CI	*p*-value	Est.	95% CI	p-value	Est.	95% CI	p-value
**DEPRESSION (CESD-10)**									
BMI Classification (ref: “normal-weight”)									
Underweight	0.01	−0.06, 0.08	.7269	−0.06	− 0.13, 0.01	.1183	− 0.06	− 0.12, 0.01	.0885
Overweight	**0.08**	**0.05, 0.11**	**<.0001**	−0.02	− 0.05, 0.01	.2173	− 0.02	− 0.05, 0.01	.1835
Obesity	**0.21**	**0.17, 0.25**	**<.0001**	0.05	0.01, 0.10	.0153	0.05	0.00, 0.09	.0345
Missing BMI	**0.08**	**0.06, 0.09**	**<.0001**	**0.04**	**0.02, 0.05**	**.0001**	**0.04**	**0.02, 0.06**	**<.0001**
Weight Perception (ref: “About the right weight”)									
Underweight				**0.19**	**0.15, 0.22**	**<.0001**	**0.16**	**0.13, 0.19**	**<.0001**
Overweight				**0.25**	**0.23, 0.28**	**<.0001**	**0.24**	**0.22, 0.26**	**<.0001**
Missing WP				**0.14**	**0.06, 0.22**	**.0006**	0.12	0.04, 0.20	.0026
Bullying Victimization in the last 30 days (ref: Yes)									
No							**−0.40**	**−0.42, − 0.38**	**<.0001**
R^2^			0.027			0.042			0.098
**ANXIETY (GAD-7)**									
BMI Classification (ref: “normal-weight”)									
Underweight	0.05	−0.04, 0.14	.2614	− 0.03	− 0.12, 0.06	.5555	− 0.04	− 0.12, 0.05	.4186
Overweight	**0.08**	**0.05, 0.11**	**<.0001**	−0.02	− 0.05, 0.01	.1315	− 0.02	− 0.06, 0.01	.1115
Obesity	**0.15**	**0.10, 0.21**	**<.0001**	0.00	−0.05, 0.06	.9356	0.00	−0.06, 0.05	.8812
Missing BMI	0.01	− 0.01, 0.03	.4542	−0.03	−0.05, − 0.01	.0053	−0.03	− 0.05, − 0.01	.0073
Weight Perception (ref: “About the right weight”)									
Underweight				**0.20**	**0.16, 0.24**	**<.0001**	**0.17**	**0.14, 0.21**	**<.0001**
Overweight				**0.25**	**0.22, 0.28**	**<.0001**	**0.23**	**0.21, 0.26**	**<.0001**
Missing WP				0.16	0.04, 0.27	.0061	0.13	0.01, 0.24	0.0270
Bullying Victimization in the last 30 days (ref: Yes)									
No							**−0.43**	**−0.45, −0.40**	**<.0001**
R^2^			0.016			0.030			0.079
**FLOURISHING (FS)**									
BMI Classification (ref: “normal-weight”)									
Underweight	−0.02	−0.05, 0.00	.0439	0.00	−0.03, 0.02	.6864	0.00	−0.03, 0.02	.7610
Overweight	**−0.02**	**−0.03, − 0.02**	**<.0001**	0.01	0.00, 0.02	.0127	0.01	0.00, 0.02	.0081
Obesity	**−0.06**	**− 0.08, − 0.05**	**<.0001**	−0.01	− 0.02, 0.00	.1422	− 0.01	−0.02, 0.01	.2078
Missing BMI	**−0.05**	**−0.06, − 0.04**	**<.0001**	**−0.04**	**− 0.04, − 0.03**	**<.0001**	**−0.04**	**− 0.04, − 0.03**	**<.0001**
Weight Perception (ref: “About the right weight”)									
Underweight				**−0.05**	**−0.06, − 0.05**	**<.0001**	**−0.05**	**− 0.06, − 0.04**	**<.0001**
Overweight				**−0.08**	**−0.09, − 0.08**	**<.0001**	**−0.08**	**− 0.09, − 0.07**	**<.0001**
Missing WP				**−0.07**	**−0.10, − 0.04**	**<.0001**	**−0.07**	**− 0.10, − 0.03**	**<.0001**
Bullying Victimization in the last 30 days (ref: Yes)									
No							**0.08**	**0.07, 0.09**	**<.0001**
R^2^			0.034			0.054			0.074

Note: All models include sociodemographic covariates (i.e., student grade and race/ethnicity, school area median household income and urbanicity) and adjust for school clustering. Est. = parameter estimate

Controlling for BMI classifications, both underweight and overweight perceptions were associated with higher depression and anxiety symptoms, and lower flourishing, relative to perceptions of being at “about the right weight”. Missing WP responses were also associated with lower flourishing scores, but not depression or anxiety symptoms. Girls who had not experienced bullying victimization in the last 30 days were at lower risk of depression and generalized anxiety symptoms, and had higher flourishing scores, than students who had been bullied in the last 30 days.

#### Boys

Table 3 presents the results of the regression models in boys. In the first three models, both obesity and missing BMI were associated with higher risk of depression and anxiety symptoms, and lower flourishing, relative to normal-weight BMI. No significant associations resulted for overweight BMI in males. Underweight BMI classifications were associated with lower flourishing relative to normal-weight BMI, but not with anxiety or depression symptoms. When WP and bullying victimization were added to the models, results for obesity and underweight BMI classifications were no longer significant, missing BMI remained associated with depression risk and lower flourishing, and a significant but negligible association emerged for overweight BMI and flourishing scores. Underweight and overweight perceptions were associated with depression and anxiety symptoms, and lower flourishing, relative to “about right” perceptions, controlling for BMI classification. Missing WP was also associated with lower flourishing scores. Boys who had not experienced bullying victimization had lower risk of depression and anxiety, and higher flourishing, relative to boys who had been bullied in the last 30 days.

**Table 3:** Weight status (BMI classification), weight perception (WP), and bullying victimization as predictors of depression, anxiety, and flourishing scores among **boys** in Year 7 (2018/19) of the COMPASS Study

	BMI			BMI + WP			BMI + WP + Bullying		
	Est.	95% CI	p-value	Est.	95% CI	p-value	Est.	95% CI	p-value
**DEPRESSION (CESD-10)**									
BMI Classification (ref: “normal-weight”)									
Underweight	0.07	0.01, 0.14	.0287	0.02	−0.04, 0.08	.5502	0.01	−0.05, 0.08	.6949
Overweight	0.01	−0.02, 0.03	.6834	−0.03	−0.06, 0.00	.0631	−0.03	− 0.06, 0.00	.0430
Obesity	**0.10**	**0.06, 0.14**	**<.0001**	0.00	−0.04, 0.04	.9324	0.00	−0.04, 0.03	.8410
Missing BMI	**0.13**	**0.11, 0.16**	**<.0001**	**0.11**	**0.08, 0.13**	**<.0001**	**0.11**	**0.09, 0.13**	**<.0001**
Weight Perception (ref: “About the right weight”)									
Underweight				**0.14**	**0.12, 0.16**	**<.0001**	**0.12**	**0.10, 0.15**	**<.0001**
Overweight				**0.19**	**0.17, 0.22**	**<.0001**	**0.17**	**0.15, 0.20**	**<.0001**
Missing WP				**0.12**	**0.05, 0.19**	**.0005**	0.09	0.02, 0.15	.0145
Bullying Victimization in the last 30 days (ref: Yes)									
No							**−0.38**	**−0.40, −0.36**	**<.0001**
R^2^			0.016			0.047			0.113
**ANXIETY (GAD-7)**									
BMI Classification (ref: “normal-weight”)									
Underweight	0.08	0.00, 0.17	.0485	0.01	−0.08, 0.09	.8855	−0.01	−0.09, 0.07	.8269
Overweight	0.01	−0.03, 0.04	.6762	−0.04	−0.08, − 0.01	.0151	−0.04	− 0.08, − 0.01	.0126
Obesity	**0.09**	**0.04, 0.14**	**.0005**	−0.06	−0.11, − 0.01	.0259	−0.06	− 0.11, − 0.01	.0125
Missing BMI	**0.05**	**0.02, 0.08**	**.0009**	0.01	−0.02, 0.04	.4800	0.01	−0.02, 0.04	.6061
Weight Perception (ref: “About the right weight”)									
Underweight				**0.21**	**0.18, 0.23**	**<.0001**	**0.19**	**0.16, 0.21**	**<.0001**
Overweight				**0.28**	**0.24, 0.32**	**<.0001**	**0.25**	**0.22, 0.29**	**<.0001**
Missing WP				−0.01	−0.10, 0.08	.8136	−0.05	−0.15, 0.04	.2581
Bullying Victimization in the last 30 days (ref: Yes)									
No							**−0.49**	**−0.53, − 0.46**	**<.0001**
R^2^			0.017			0.037			0.091
**FLOURISHING (FS)**									
BMI Classification (ref: “normal-weight”)									
Underweight	**−0.04**	**−0.06, −0.03**	**<.0001**	−0.03	−0.05, − 0.01	.0021	−0.03	− 0.04, − 0.01	.0031
Overweight	0.00	−0.01, 0.00	.4782	0.01	0.00, 0.02	.0016	**0.01**	**0.01, 0.02**	**.0009**
Obesity	**−0.04**	**−0.05, − 0.03**	**<.0001**	0.00	−0.01, 0.01	.6217	0.00	−0.01, 0.02	.4756
Missing BMI	**−0.07**	**−0.08, − 0.06**	**<.0001**	**−0.06**	**− 0.07, − 0.05**	**<.0001**	**−0.06**	**− 0.06, − 0.05**	**<.0001**
Weight Perception (ref: “About the right weight”)									
Underweight				**−0.04**	**−0.05, − 0.04**	**<.0001**	**−0.04**	**− 0.05, − 0.03**	**<.0001**
Overweight				**−0.07**	**−0.08, − 0.06**	**<.0001**	**−0.07**	**− 0.08, − 0.06**	**<.0001**
Missing WP							**−0.05**	**−0.07, − 0.03**	**<.0001**
Bullying Victimization in the last 30 days (ref: Yes)									
No							**0.08**	**0.07, 0.09**	**<.0001**
R^2^			0.027			0.059			0.084

Note: All models include sociodemographic covariates (i.e., student grade and ethnicity, school area median household income and urbanicity) and adjust for school clustering. Est. = parameter estimate

While the variance explained by all models was low, the models explained progressively more variance with the addition of weight perception to models, and then with the addition of bullying. The increase in the variance explained with the addition of bullying was comparable to (e.g., Flourishing models) or slightly greater than (e.g., Anxiety models) the increase with the addition of weight perception.

## Discussion

We sought to determine if WP and bullying victimization account for links between weight status and mental health outcomes, and if relationships differed by gender, in a large population sample of Canadian secondary school students. We found that perceptions of deviating from “about the right weight” and having experienced bullying victimization independently predicted depression and anxiety symptoms, and poorer psychosocial well-being, when weight status and sociodemographic covariates were controlled for. Results support our hypotheses and existing literature suggesting that the mental health risks associated with overweight and obesity BMI are largely negated when controlling for WP and bullying victimization [10, 29, 30, 38]. That is, individuals’ perceptions of their weight and experiences of bullying appear to account for the detrimental mental health effects associated obesity, rather than body weight itself.

Results align with a recent meta-analysis in which perceived overweight status was associated with increased risk of depression and suicidality, and overweight status was no longer associated with depressive symptoms when WP was added to the predictive model [29]. The authors concluded that the detrimental effect of overweight on mental health is largely dependent on whether a person identifies as overweight [29]. Similarly, a longitudinal study of Dutch youth found that perceptions of overweight, but not measured or self-reported weight status, predicted poorer mental health [26]. Likewise, in a large cross-sectional study of US youth in grades 8, 9, and 11, those who perceived themselves as overweight across BMI categories, were more likely to experience high levels of internal distress and lower psychosocial protective factors (parent, friend, and school connectedness, positive identity, social competency) than those who had “about right” weight perceptions [38].

Overweight perceptions may present mental health risks through the internalization of weight bias; that is, believing the stigma associated with larger body sizes applies to oneself. In the literature, WP is typically compared to one’s BMI weight status to classify individuals as having an accurate or misperceived WP. However, previous research suggests that misperceptions are not the concern; overweight and underweight perceptions appear detrimental across BMI categories [38, 69]. Moreover, despite being the most used indicator of WP, how youth interpret the single item measure is not entirely clear. Adolescents may respond by comparing their weight to a medical standard such as BMI, or to their ideal body, their peers, or some other alternative. It is plausible that responses of “about the right weight” indicate weight satisfaction rather than youths’ perception of how their weight compares to an external reference point. Body dissatisfaction has been linked to nonresponse to weight and height measures [68, 70], and interestingly, both boys and girls with missing BMI data reported greater depression symptoms and lower flourishing relative to their peers with normal-weight BMIs, and unlike results for overweight and obesity, the associations remained significant while controlling for WP and bullying victimization. It is plausible that body dissatisfaction may explain poor mental health outcomes in individuals with non-response to height and weight survey items. However, in a US study of 11–17-year olds, overweight perceptions, and not body dissatisfaction, increased risk of major depression, regardless of measured BMI [30].

While most research has focused on overweight perceptions, this study provides further evidence that perceptions of deviating from “about the right weight” in either direction have detrimental associations with youth health [23, 26, 39]. As expected, underweight perceptions were more common in boys than girls; over one-fifth of boys and one-tenth of girls reported perceptions of underweight, despite less than 2% having BMIs classified as underweight. However, in contrast to our hypothesis, associations between underweight perceptions and increased depression and anxiety, and lower flourishing, were comparable across boys and girls. Previous studies have found perceptions of underweight to be associated with depression and anxiety in males [24, 44, 45], and increased odds of psychological distress, internalizing symptoms, and suicidality in all youth [24, 25, 44, 45]. In a recent UK longitudinal study, overweight perceptions in females only, and underweight perceptions in both males and females, predicted clinically relevant internalizing symptoms among adolescents with normal-weight BMIs [39]. Our results align with findings for underweight perceptions, but we found overweight perceptions to present risk in both boys and girls for increased internalizing symptoms. Replication of results using prospective designs is necessary.

Experiences of bullying victimization were associated with greater depression and anxiety symptoms and lower flourishing scores in both males and females, independent of WP and weight status. Several studies have demonstrated bullying as predictor of poor psychosocial health and psychopathology [71–73]. Consistent with previous reports [16], bullying victimization was reported more often among boys and girls with BMIs in the obesity range relative to their peers with normal-weight BMIs; although reports of bullying victimization were most frequent among boys with underweight BMIs, and equivalent in girls with BMIs in the underweight and obesity ranges. A u-shaped association between body weight and bullying victimization was previously found in male but not female adolescents [74].

One plausible mechanism contributing to the associations found is WP and bullying victimization may deter engagement in behaviours that promote mental health and prevent or manage internalizing symptoms. Increasing awareness among individuals of their weight status—or “correcting” so-called weight “misperceptions”—has been rationalized as a means to motivate healthy behaviours, but overweight perceptions are associated with unhealthy weight-control behaviours [75], more sedentary time, lower physical activity engagement [69], less healthy dietary behaviours [69], and poor academic achievement [76], regardless of BMI classification. Similarly, over a one-year period, underweight perceptions predicted lower engagement in various types of physical activity in boys and girls, and less healthful dietary intake in girls, than their peers with about right perceptions [69]. Bullying victimization also predicts greater engagement in risk behaviours, including substance use [16], disordered eating behaviour [77], and nonadherence to the 24-h movement behaviour guidelines [78]. Therefore, WP and bullying victimization may indirectly influence poor mental health via their effects on engagement in health promotive behaviours.

Results highlight the need to screen for depression and anxiety among adolescents exposed to bullying victimization, and the importance of bullying prevention in mental health promotion and mental illness prevention strategies targeting adolescent populations. Despite vast attention to bullying over the past decade, rates remained relatively stable, particularly among girls [79]. Previous research found support for emotional dysregulation and low self esteem as contributors to links between bullying victimization and internalizing symptoms among adolescents [73, 80, 81]; hence, upstream approaches to promote healthy socioemotional skills have been suggested to both prevent bullying involvement and help protect students against the impact of bullying on their mental health [73]. Interventions targeting weight bias and promoting weight acceptance may prove valuable to prevent weight-based bullying and to rectify the negative connotations of varying body sizes.

Bullying prevention may also prevent underweight or overweight perceptions [74]. Several cross-sectional studies demonstrate an association between bullying victimization and either overweight or underweight perceptions [48, 82–86], and in a large prospective adolescent sample, experiences of bullying were found to predict changes from “about the right weight” to overweight perceptions [86]. It is plausible that bullying victimization, leads to changes in weight perception and/or internalized weight stigma, and in turn, poor mental health. In support, Lee and Vaillancourt argue bullied adolescents blame themselves and come to see themselves as internally flawed, particularly in terms of their bodies, leading to internalizing symptoms [77]. Upstream approaches are needed to promote size acceptance and deconstruct the stigma surrounding body weight that contributes to the negative connotations and experiences associated with body sizes outside of “normal weight”, including the dominant obesity narratives portrayed in public health, education, and the media [35, 87].

This study has several strengths, including the large adolescent sample from four Canadian provinces, examination of gender differences, and the inclusion of both underweight and overweight perceptions, and both psychopathology and mental well-being. That said, this study is not without limitations. BMI was based on self-reported height and weight, and does not measure body composition or distribution. As such, results likely reflect greater concordance between weight perception and weight status than exists. However, a strong correlation between measured and self-reported BMI has been found in youth [54, 88, 89]. The weight status measure has been found to be reliable and valid for use among large youth populations when objective methods are not feasible or ethical due to potential harm in school-based settings [67]. Furthermore, results align with previous studies of WP and mental health using measured BMI [10, 25]. Students missing weight perception or BMI data were retained in the models, but the removal of students missing outcome data presents a limitation. Students not responding to the mental health measures may be at higher risk of poor mental health or depression/anxiety. The use of passive consent protocols for depression screenings is shown to better reach students at risk of depression [50]. Also, the COMPASS study does not require student names, helping to preserve perceptions of anonymity for honest reporting. The bullying measure referred to general experiences of bullying and does not specify whether bullying was related to weight. Also, as only bullying in the last 30 days was assessed, individuals experiencing bullying outside of this time might not have been captured. The primarily limitation is the cross-sectional design. Future analysis of the longitudinal COMPASS study will allow for examination of weight perception trajectories and the relationship to mental health outcomes over time. Measures only allowed examination of cisgender students. Lastly, COMPASS uses a convenience sample of schools and was not designed to be representative, although the study size, whole school samples, and favourable response rates support generalizability.

## Conclusion

Results support previous literature indicating adolescents’ perceptions of their weight and experiences of bullying victimization account for the associations between obesity and poor psychosocial well-being and internalizing symptoms. Regardless of weight status, perceptions of overweight or underweight were associated with greater depression and anxiety symptomatology, and poorer flourishing scores, relative to perceptions of being at “about the right weight”. Likewise, youth who had experienced bullying victimization within the past month had greater risk of poor mental health and internalizing symptoms. Findings suggest enhanced investment in bullying prevention, and the promotion of body size acceptance and positive body image, may prove beneficial for the prevention of mental illness and promotion of mental well-being among youth of all body sizes. Further research is needed to examine relationships prospectively to establish temporality and explore mechanisms in the relationships identified.

## Data Availability

COMPASS study data is available upon request through completion and approval of an online form: https://uwaterloo.ca/compass-system/information-researchers/data-usage-application. The datasets used during the current study are available from the corresponding author on reasonable request.

## Abbreviations

BMI: Body Mass Index
CESD-10: 10-item Center for Epidemiologic Studies Depression scale Revised
COMPASS: Cannabis, Obesity, Mental health, Physical activity, Alcohol, Smoking, and Sedentary behaviour
GAD-7: Generalized Anxiety Disorder Scale
Est.: Parameter estimate
FS: Flourishing Scale
WP: Weight perception
